# The contribution of snacks to dietary intake and their association with eating location among Norwegian adults – results from a cross-sectional dietary survey

**DOI:** 10.1186/s12889-015-1712-7

**Published:** 2015-04-12

**Authors:** Jannicke B Myhre, Elin B Løken, Margareta Wandel, Lene F Andersen

**Affiliations:** Department of Nutrition, University of Oslo, P.O.Box 1046 Blindern, 0317 Oslo, Norway

**Keywords:** Snack, Meal, Energy intake, Norway, Dietary survey, Eating location, Adult

## Abstract

**Background:**

Snack consumption has been reported to increase over recent decades. Little is known about possible associations between snack composition and snack eating location. In the present study, we aimed to describe the contribution of snacks to dietary intake in Norwegian adults and to investigate whether the composition of snacks differed according to where they were eaten.

**Methods:**

Dietary data were collected in 2010 and 2011 using two telephone administered 24 h recalls about four weeks apart. In total, 1787 participants aged 18-70 years completed two recalls. The recorded eating locations were at home, other private household, work/school, restaurant/cafe/fast-food outlet and travel/meeting.

**Results:**

Snacks contributed to 17% and 21% of the energy intake in men and women, respectively. Compared with main meals, snacks had a higher fiber density (g/MJ) and contained a higher percentage of energy from carbohydrates, added sugars and alcohol, while the percentages of energy from fat and protein were lower. The top five energy-contributing food groups from snacks were cakes, fruits, sugar/sweets, bread and alcoholic beverages. Snacks were mostly eaten at home (58% of all snacks) or at work/school (23% of all snacks). Snacks consumed at work/school contained less energy, had a higher percentage of energy from carbohydrates and had lower percentages of energy from added sugars, alcohol and fat than snacks consumed at home. Snacks consumed during visits to private households and at restaurants/cafe/fast-food outlets contained more energy, had a higher percentage of energy from fat and had a lower fiber density than snacks consumed at home.

**Conclusions:**

We conclude that snacks are an important part of the diet and involve the consumption of both favorable and less favorable foods. Snacks eaten at home or at work/school were generally healthier than snacks consumed during visits to other private households or at restaurants/cafe/fast-food outlets. Nutritional educators should recommend healthy snack options and raise awareness of the association between eating location and snack composition.

## Background

Both the frequency of snack consumption [1] and the contribution of snacks to the total energy intake [1-3] have been reported to increase over recent decades. Studies from the USA [1,3], Canada [4] and Brazil [5] show that 21-24% of the total energy intake is derived from snacks. In Finland, the percentage of the total energy intake that is consumed as snacks has been found to be as high as 36-40% [2]. These figures are not necessarily directly comparable, as different researchers have used different dietary assessment methods and different definitions of meals, snacks and eating events. Nevertheless, it seems to be well established that snacks constitute an important part of the modern diet. Concern has been raised regarding the quality of snacks and their contribution to the total energy intake and to the overall quality of the diet. The impact of snacks on the quality of the diet will naturally depend on the composition of the snacks. Energy dense foods such as sweets, desserts, salty snacks and sugar-sweetened beverages have often been reported to be main constituents of snacks [1,2,5,6]. With regard to macronutrient composition, snacks have been found to be higher in carbohydrates and sugars but lower in fat and protein than main meals [6,7]. Snacks have also been found to contribute valuable components such as fruits [8] and micronutrients [9] to the diet. In Finland, only a few dietary differences were observed between participants with a snack-dominated meal pattern and participants with a main meal pattern. Therefore, the authors concluded that main meals and snacks are parallel ways of composing the diet with only a few dietary differences [10]. The location of snack consumption is a factor that may influence the composition of snacks. Limited research has been conducted with a specific focus on where snacks are consumed; however, a number of studies have examined the nutritional impact of out-of-home eating in general. These studies have often shown that eating outside of the home has a negative impact on the nutritional quality of the diet [11-13]. Given that snacks form such a substantial part of the total energy intake, further studies regarding the association between eating location and snack composition are warranted. The role of snacks in the Norwegian diet has not been previously studied. In the present study, we aimed to describe the contribution of snacks to dietary intake in Norwegian adults and to investigate whether the composition of snacks differed according to eating location.

## Methods

### Subjects and design

Data for the present study were obtained from Norkost 3, a dietary survey among Norwegian adults that was conducted in 2010 and 2011. The design and methodology have been described in detail elsewhere [14,15]. A representative sample (n = 5000) of the Norwegian population aged 18-70 years was randomly selected from the National Register and asked to complete two telephone-administered 24 h recalls approximately 4 weeks apart. Data were collected about all days of the week. Of the 5000 individuals who were invited to participate, 153 were unsuitable (wrong phone number, not Norwegian or invited to participate twice by mistake). Of the remaining 4847 suitable invitees, 2275 declined to participate, 530 could not be contacted, 178 agreed to participate but did not respond to subsequent phone calls and 77 completed only one 24 h recall. Thus, in total, 1787 participants successfully completed the two 24 h recalls, which resulted in a participation rate of 37%. All 1787 participants were included in the comparison of macronutrient intake from snacks and main meals and in the determination of the five food groups contributing most to the energy intake from snacks. Because information on some of their background variables was missing, 34 participants were excluded from the analyses of participants who consumed or did not consume the top five energy-contributing food groups from snacks according to BMI and educational level. Similarly, 126 snacks consumed by 33 participants were excluded from the analyses of the differences in snack composition according to eating location because information on background variables was missing. In addition, 185 snacks that were consumed by 131 persons in the locations “other” or “unknown” were excluded from the eating location analyses due to the low number of observations and the possible diverse nature of these eating locations. The study was conducted according to the guidelines established in the Declaration of Helsinki, and all procedures that involved human subjects were approved by the Regional Committee for Medical Research Ethics. Verbal informed consent was obtained from all subjects.

### Assessment of dietary intake

The 24 h recalls aimed to include all foods and beverages that were consumed by the participants from the time they awoke on the preceding day to the time they awoke on the day of the interview. The interviews were performed by trained personnel using an in-house data program (KBS version 7.0) linked directly to a food composition database. This food composition database was based on the Norwegian Food Composition Table from 2006 [16] and was supplemented with additional food items from reliable sources. A total of 2888 food items were included in the database used for the calculations in the present study. Before the recall began, the participant was asked if he or she considered the previous day to be a normal day with regard to food and beverage intake (yes/no). The interviews were conducted in a three-step process. The first step involved a review of the previous day’s eating and drinking events including the time and location of the eating/drinking event and a brief description of the foods and/or beverages that were consumed. Each eating or drinking event was defined by the respondent as either breakfast, lunch, dinner, supper/evening meal or snack. Eating events labeled as snacks might consist of only a beverage. The predetermined eating locations were “home”; “other private household”; “work or school, including work/school canteens” (hereafter called “work” due to the adult study population); “restaurants, cafés, fast food outlets” (hereafter called “restaurant”); “meeting, travel, during exercise” (hereafter called “travel/meeting”); “other location”; or “unknown location”. The eating location was defined as the place of consumption irrespective of the place of purchase or preparation. The second step of the recall involved the collection of detailed information about the food and portion sizes. The amounts of food consumed were quantified based on household measures and a booklet that contained photographs of foods in different portion sizes. The third step consisted of a checklist of commonly forgotten food items. This checklist included foods that were typically thought to be consumed between meals (i.e., as snacks) such as chewing gum, coffee, tea, water, fruits, sweet bakery products and dietary supplements. All food items labeled as dietary supplements were excluded from the analyses.

### Description of snacks and snack consumers

For the comparison of the nutrient composition of main meals and snacks the mean energy and nutrient intakes from the two 24 h recalls, including both weekdays and weekend days, were calculated. For the quantification of the number of snacks per day, only snacks that consisted of at least 50 kJ were included. This limit was set to exclude snacks with minimal contribution to nutrient intake such as those that consisted of only water, unsweetened coffee or tea, sugar-free chewing gum or sugar-free pastilles. The 50 kJ limit was also used for the enumeration of snacks that contained the primary energy-contributing food groups and for the enumeration of participants who had consumed these food groups. Finally, the 50 kJ limit was used for the comparisons of snacks that were consumed in different locations. For the estimation of macronutrient intakes and the five main energy-contributing food groups from snacks, all snacks were included, regardless of energy content.

For the determination of the top five food groups that contributed to the energy intake from snacks, the mean two-day energy contribution in kJ from snacks from the following 23 food groups was calculated: bread (including regular bread, rolls, crisp bread, crackers and tortillas); rice and pasta; breakfast cereal; other cereal products (including flour, grains, pasta dishes and pies); cakes (including buns, muffins, waffles, cookies, cream cakes and other cakes); potatoes (including boiled, fried or mashed potatoes and French fries); vegetables (all kinds of vegetables including legumes); fruits and berries (all fresh fruits and berries hereafter called “fruits”); jams and canned fruits; nuts and olives; juice (fruit/vegetable); meat and meat products; fish and fish products (including shellfish); eggs; milk; yogurt; cheese; ice cream and milk-based desserts; butter, margarine and oil (including dressing and mayonnaise-based sandwich spreads); sugar-sweetened beverages; alcoholic beverages; sugar/sweets (e.g., sugars, syrup, honey, chocolates, sweets); and salty snack items (including potato chips, pop corn, nachos and other salty chips).

The participants were defined as a consumer of each the top five food groups that contributed to the energy intake from snacks if the food group had been consumed as snack at least once during the two recall days.

### Background variables

The participants were categorized into three age groups: 18-34 years, 35-54 years and 55-70 years. The BMI was calculated based on self-reported weight and height as the weight (kg) divided by the square of the height (m^2^), and dichotomized into “normal weight (BMI < 25.0 kg/m^2^)” and “overweight (BMI ≥ 25.0 kg/m^2^).” The level of education was originally divided into eight categories that ranged from “no education” to “university/college education at masters/PhD level” but was divided into two categories: “high school, technical school, trade school or less” and “university or college education.” Smoking habits were originally grouped into three categories but were regrouped into two categories: “smoker (daily/occasional smokers)” and “non-smokers (never-smokers and previous smokers).” Interest in a healthy diet was originally grouped into five categories that ranged from “no interest” to “very high interest” but was regrouped into two categories: “no, low or moderate interest” and “high or very high interest.” Weekdays were defined as Monday to Friday, while weekend days were defined as Saturday and Sunday. Participants were categorized as under-reporters with regard to energy intake if their estimated energy intake (EI) from the 24 h recalls divided by their estimated basal metabolic rate (BMR) (EI/BMR) was lower than 0.96 [17,18]. Sixteen percent of the participants were categorized as under-reporters [15].

### Statistical analyses

Statistical analyses were performed with Stata version 13.1 (StataCorp LP, Texas, USA). All tests were two-sided. For the comparisons of energy intake and the intake of macronutrients from snacks and main meals, the paired samples *T*-test was used. These analyses were conducted separately for men and women. The results are presented as means and 95% confidence intervals. For the comparison of the number of snacks consumed per day in men and women according to age, BMI and educational level, linear regression was used. Differences in the percentage of consumers of the five main energy contributing food groups from snacks according to BMI and educational level were analyzed using logistic regression with consumption/no consumption of each of the food groups as the dependent variable and the categorical variables of gender, age group, BMI, educational level, smoking habits, interest in a healthy diet and whether the participant was an under-reporter of energy as independent variables. The results are presented as the percentages of participants who consumed the respective food groups. For the comparison of energy and macronutrient intakes as well as consumption of the five food groups according to eating location, repeated observations were available for the majority of the participants because of consumption of more than one snack during the two recall days. Mixed models were used to adjust for this dependency in the data via the addition of a variance component (random intercept) for each participant. Linear mixed models were used for the continuous variables, while a logistic mixed model was used for the dichotomized variables (consumer or non-consumer of each food group). For the mixed model analyses involving energy and macronutrients, case bootstrapping with 1000 repetitions was applied due to the large number of zeros in the data, particularly for alcohol (the majority of snacks did not contain alcohol). To retain the dependency structure in the bootstrap samples, participants rather than individual observations were sampled. The mixed models were adjusted for the categorical variables of gender, age group, BMI, educational level, smoking habits, interest in a healthy diet, weekday/weekend day, if the day was a normal day or not with regard to food and beverage intake and whether the participant was an underreporter of energy. The results from the linear mixed models are presented as adjusted means, bootstrap 95% confidence intervals and bootstrap p-values. The results from the logistic mixed models are presented as percentages of snacks that contained each of the food groups and p-values. Because 12 tests were conducted for each eating location in, the significance level was adjusted to p < 0.004 (p < 0.05 divided by 12 tests). For all other analyses, a significance level of p < 0.05 was chosen.

## Results

### The contribution of snacks to the total dietary intake

Table 1 shows the background characteristics and the number of snacks consumed per day for the participants in the Norkost 3 study. The mean number of snacks was 1.6 per day for men (range 0-11) and 1.9 per day for women (range 0-10). A total of 93% of men and 97% of women had consumed at least one snack during the two recall days, and snacks contributed to 17% and 21% of the total energy intake among men and women, respectively. Collectively for men and women, snacks contributed 19% of energy intake, 11% of protein intake, 16% of fat intake, 22% of carbohydrate intake, 40% of the intake of added sugars, 20% of fiber intake and 48% of alcohol intake.

**Table 1:** **Background characteristics of the participants, Norkost 3 study, 2010-2011 (n = 1787)**

	**Men**			**Women**		
	**Norkost 3 n = 862**		**Snacks per day** ^**a**^	**Norkost 3 n = 925**		**Snacks per day** ^**a**^
Age group, n = 1787	n	%		n	%	%
18-34 years	199	23.1	1.6	208	22.5	1.9
35-54 years	355	41.2	1.6	461	49.8	1.9
55-70 years	308	35.7	1.5	256	27.7	1.8
BMI, n = 1756						
<25.0 kg/m^2^	344	40.0	1.7	544	60.8	1.9
≥25.0 kg/m^2^	517	60.0	1.5**	351	39.2	1.8*
Educational level, n = 1784						
High school or less	432	50.2	1.4	414	44.9	1.8
University or college	429	49.8	1.7***	509	55.1	2.0**

BMI, body mass index.

^a^Only snacks ≥50 kJ are included.

*p < 0.05, linear regression that compares the number of snacks per day in men and women separately; the first category is used as a reference category for each of the background characteristics.

**p < 0.01, linear regression.

***p < 0.001, linear regression.

Table 2 shows a comparison of the macronutrient composition of snacks and main meals by gender. For both men and women, snacks were different from main meals with regard to the percentage of energy that was derived from each of the macronutrients. Snacks had lower percentages of energy from fat and protein and a higher percentage of energy from carbohydrates, added sugars and alcohol. Snacks also had a higher fiber density than main meals. To see if the differences in nutrient composition between snacks and main meals were driven by different snacks on weekend days versus weekdays, the analyses were also run including only participants with both recalls covering weekdays (n = 1000). However, for both men and women the p-values remained the same (p < 0.001) and only minor differences in the percentages of energy from each of the macronutrients (data not shown) were seen compared to the analyses including all days of the week.

**Table 2:** **Energy and macronutrient intakes from snacks and main meals (n = 1787)**

	**Men (n = 862)**					**Women (n = 925)**				
	**Snacks**		**Main meals** ^**a**^		**p** ^**b**^	**Snacks**		**Main meals** ^**a**^		**p** ^**b**^
	**Mean**	**95% CI**	**Mean**	**95% CI**		**Mean**	**95% CI**	**Mean**	**95% CI**	
Energy, MJ	1.8	1.7,1.9	9.0	8.9,9.2	<0.001	1.7	1.6,1.8	6.3	6.2,6.4	<0.001
Fat, E%	24	23,25	35	35,36	<0.001	27	26,28	35	35,36	<0.001
Protein, E%	13	12,14	19	19,19	<0.001	11	10,12	19	19,20	<0.001
Carbohydrate, E%	52	51,54	42	41,42	<0.001	53	52,54	42	41,42	<0.001
Added sugars, E%	16	15,17	5	5,6	<0.001	15	14,16	5	5,6	<0.001
Fiber, g/MJ	3.2	3.0,3.4	2.5	2.4,2.5	<0.001	3.5	3.3,3.6	2.8	2.7,2.9	<0.001
Alcohol, E%	5	4,6	2	1,2	<0.001	4	3,5	1	1,2	<0.001

MJ, mega joule; E%, percentage of energy.

^a^Breakfast, lunch, dinner and supper/evening meal.

^b^Comparison of the percentage of energy derived from each macronutrient, paired samples *T*-test.

**Figure 1:**
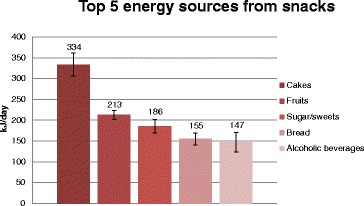
Top five sources of energy (kJ/day, 95% CI) from snacks, Norkost 3 study, 2010-2011 (n = 1787). “cakes” include buns, muffins, waffles, cookies, cream cakes and other cakes, “fruits” include fresh fruits and berries, “sugar/sweets” include sugars, syrup, honey, chocolates, and sweets, “bread” includes regular bread, rolls, crisp bread, crackers and tortillas.

Figure 1 illustrates the five food groups that contributed the most to the energy intake from snacks. On average, these five food groups contributed with 59% of the total snack energy intake. Cakes contributed the most to energy intake from snacks and were included in 15% of all snacks (Table 3). Cakes were consumed as snacks by 38% of the participants (Table 4). Fruits were the second largest contributor to the energy intake from snacks, and 38% of all snacks contained fruits. Sixty-eight percent of the participants reported consuming a snack that contained fruits. The third, fourth and fifth largest contributors to the energy intake from snacks were sugar/sweets, bread and alcoholic beverages.

**Table 3:** **Inclusion of cakes, fruits, sugar/sweets, bread and alcoholic beverages in 6188 snacks** ^**a**^ **(n = 1787)**

**Food group** ^**b**^	**Snacks that contained the food group with or without other foods (n = 6188 snacks)** ^**a**^	
	**n**	**%**
Cakes	929	15
Fruits	2355	38
Sugar/sweets	1616	26
Bread	852	14
Alcoholic beverages	442	7

^a^Only snacks ≥50 kJ are included.

^b^“cakes” include buns, muffins, waffles, cookies, cream cakes and other cakes, “fruits” include fresh fruits and berries, “sugar/sweets” include sugars, syrup, honey, chocolates, and sweets, “bread” includes regular bread, rolls, crisp bread, crackers and tortillas.

**Table 4:** **Consumer proportion for cakes, fruits, sugar/sweets, bread and alcoholic beverages in snacks** ^**a**^ **in all participants (n = 1787) and according to BMI and education (n = 1753)**

	**Percentage of participants who consumed the food group as a snack** ^**a**^						
**Food group** ^**b**^	**All participants n = 1787**	**BMI**			**Educational level**		
		**BMI < 25 kg/m** ^**2**^ **n = 888**	**BMI ≥ 25 kg/m** ^**2**^ **n = 865**	**p** ^**c**^	**High school or less n = 829**	**University/college n = 924**	**p** ^**c**^
	**%**	**%**	**%**		**%**	**%**	
Cakes	38	38	38	0.23	35	41	0.036
Fruits	68	71	65	0.053	62	73	<0.001
Sugar/sweets	57	61	52	0.25	55	58	0.82
Bread	35	37	33	0.97	35	35	0.33
Alcoholic beverages	18	20	16	0.025	17	19	0.17

BMI, body mass index.

^a^Only snacks ≥50 kJ are included.

^b^“cakes” include buns, muffins, waffles, cookies, cream cakes and other cakes, “fruits” include fresh fruits and berries, “sugar/sweets” include sugars, syrup, honey, chocolates, and sweets, “bread” includes regular bread, rolls, crisp bread, crackers and tortillas.

^c^Logistic regression (consuming or not consuming each food group as a snack was used as a dichotomous dependent variable) adjusted for gender, BMI, educational level, age group, interest in a healthy diet, smoking habit and whether the participant was characterized as an under-reporter of energy intake.

Table 4 shows the percentage of participants who consumed at least one snack containing cakes, fruits, sugar/sweets, bread or alcoholic beverages for all participants and according to BMI and educational level. A higher percentage of participants with university/college education reported consuming a snack that contained fruits compared with participants without university/college education (73% vs. 62%, p < 0.001). With regard to BMI, the percentage of participants who consumed alcoholic beverages was lower in the overweight group compared with the group of participants with a BMI < 25 kg/m^2^ (16% vs. 20%, p = 0.025).

### Snack eating location

Table 5 shows the content of energy and macronutrients in snacks that were consumed in different locations. This table also shows the percentages of snacks that included cakes, fruits, sugar/sweets, bread and alcoholic beverages for each eating location. The majority of snacks were eaten at home (58%) and at work (23%). As many as 87% of the participants reported consuming at least one snack at home, while 45% reported consuming at least one snack at work. The energy intake from snacks that were consumed at work (0.7 MJ/snack) was lower than from snacks that were eaten at home (1.0 MJ/snack), while snacks that were eaten during visits to private households (1.7 MJ/snack) and at restaurants (1.3 MJ/snack) contained more energy than snacks that were consumed at home. Snacks that were consumed at work had a higher percentage of energy from carbohydrates and contained more fiber per MJ, while the percentages of energy from fat, alcohol and added sugars were lower than those for snacks consumed at home. In contrast, snacks consumed at restaurants and during visits to private households had a higher percentage of energy from fat, while the percentage of energy from carbohydrates and the fiber intake in g/MJ were lower compared to snacks consumed at home. Snacks consumed during visits to other private households also had a higher percentage of energy from added sugars than snacks at home. Restaurant snacks had a higher percentage of energy from alcohol than snacks consumed at home.

**Table 5:** **Intake of energy, macronutrients and selected food groups in snacks** ^**a**^ **eaten at different locations (n = 1661)**

	**Home**		**Visiting private households**			**Work** ^**b**^			**Restaurant** ^**c**^			**Travel/meeting** ^**d**^		
Number (%) of snacks (n = 5876 snacks) consumed in each location	3396 (58%)		336 (6%)			1376 (23%)			195 (3%)			573 (10%)		
Number (%) of participants consuming ≥1 snack in this location (n = 1661)	1438 (87%)		258 (16%)			745 (45%)			154 (9%)			394 (24%)		
Energy and macronutrients	Mean^e^	95% CI	Mean^e^	95% CI	p^f^	Mean^e^	95% CI	p^f^	Mean^e^	95% CI	p^f^	Mean^e^	95% CI	p^f^
Energy, MJ/snack	1.0	1.0,1.0	1.7	1.6,1.9	<0.001	0.7	0.7,0.8	<0.001	1.3	1.2,1.5	<0.001	1.0	0.9,1.1	0.97
Protein, E%	10	10,10	9	8,10	0.012	11	10,12	0.005	10	9,11	0.76	10	9,11	0.70
Fat, E%	23	22,23	30	28,33	<0.001	19	18,21	<0.001	29	26,33	<0.001	23	21,25	0.62
Carbohydrates, E%	59	58,60	52	50,55	<0.001	64	63,66	<0.001	44	42,47	<0.001	62	60,64	0.008
Added sugars, E%	15	14,16	22	19,24	<0.001	10	9,11	<0.001	16	14,19	0.37	19	17,22	0.001
Fiber, g/MJ	4.1	3.9,4.2	2.4	2.0,2.7	<0.001	6.0	5.7,6.3	<0.001	1.5	1.1,1.9	<0.001	3.9	3.5,4.4	0.55
Alcohol, E%	5	5,6	7	4,9	0.25	0	0,1	<0.001	15	11,19	<0.001	2	0,3	<0.001
Food groups^g^	%		%		p^h^	%		p^h^	%		p^h^	%		p^h^
Cakes, % of snacks including	12		43		<0.001	10		0.68	29		<0.001	20		<0.001
Fruits, % of snacks including	37		27		0.003	49		<0.001	12		<0.001	35		0.81
Sugar/sweets, % of snacks including	27		34		0.023	20		<0.001	26		0.44	30		0.20
Bread, % of snacks including	12		13		0.44	19		<0.001	15		0.11	12		0.91
Alcoholic beverages, % of snacks including	9		12		0.06	0		<0.001	27		<0.001	4		<0.001

MJ, mega joule; E%, percentage of energy.

^a^Only snacks ≥50 kJ are included.

^b^Work and school, including school/work canteens

^c^Restaurant, fast food outlet, café

^d^Travel, meeting, during exercise

^e^Means adjusted for gender, age group, BMI, educational level, interest in a healthy diet, smoking habits, weekday/weekend day, normal day or not with regard to food and beverage intake and whether the participant was an under-reporter of energy intake on the day the snack was consumed.

^f^Linear mixed models with bootstrap; the eating location “home” is used as a reference.

^g^“cakes” include buns, muffins, waffles, cookies, cream cakes and other cakes, “fruits” include fresh fruits and berries, “sugar/sweets” include sugars, syrup, honey, chocolates, and sweets, “bread” includes regular bread, rolls, crisp bread, crackers and tortillas.

^h^Logistic mixed models; the eating location “home” is used as a reference.

Due to multiple comparisons, the significance level was set to p < 0.004.

With regard to the use of the top five energy- contributing food groups as snacks, more snacks consumed at work contained fruits or bread, while fewer contained sugar/sweets or alcoholic beverages than snacks consumed at home. For snacks that were consumed during visits to private households or at restaurants, cakes were more common while fruits were less common compared with snacks consumed at home. For restaurant snacks, consumption of alcoholic beverages was also more common than for snacks eaten at home.

## Discussion

### The contribution of snacks to the total dietary intake

The present study showed that a considerable proportion of daily energy intake was derived from snacks: 17% for men and 21% for women. These results are in the lower range of published results from studies conducted in other countries [3-5,7]. Our results are also markedly lower than what was found in a Finnish study conducted in 2002 in which 36% and 40% of energy intake came from consumption of snacks among men and women, respectively [2]. One explanation for the relatively low energy intake from snacks in our study compared with other studies is the inclusion of supper/evening meal as a main meal rather than as a snack. Hence, we have chosen to use a four-meal pattern rather than the three-meal pattern, which is used in most Western countries. This approach was used because supper/evening meal is a commonly consumed meal in Norway [8,19,20]. As previously reported [8], the evening meal contained significantly less fruits and more whole grains than snacks. Also, the intakes of cakes, sugar/sweets and alcoholic beverages were lower from the evening meal while the intake of bread was higher than from snacks (data not shown). If supper/evening meal were regarded as a snack, 29% of energy would be derived from snacks for men and 31% of energy would be derived from snacks for women.

Similar to other studies, we found that snacks contained a higher percentage of energy from carbohydrates and lower percentages of energy from fat and protein compared with main meals [6,7]. The percentage of energy from added sugar was also considerably higher from snacks than from main meals. The differences between snacks and main meals were maintained also when looking at participants having recalls covering weekdays only, indicating that differences between snacks and main meals are not only occurring during the weekends. An association between sugar intake and snacks has also been reported by others [6,10,21]. In the present study, 40% of the total intake of added sugars came from snacks. Both cakes and sugar/sweets were among the top five energy-contributing food groups from snacks; these are foods that contain quite high amounts of added sugar. Hence, replacement of some sugar dense foods with healthier snack options could contribute to a considerable reduction in sugar intake. However, we also found that snacks had a somewhat higher fiber density (in g/MJ) than main meals. The amount of fiber provided by snacks has not frequently been reported in the literature. However, in Finland, women with a snack-dominated meal pattern had lower fiber intakes than women with a main meal- dominated pattern, which suggests that snacks might contain less fiber than main meals [10]. On the contrary, results from the US National Health and Nutrition Examination Survey (NHANES) from 1988-94 showed that eating frequency was positively associated with fiber intake after adjustment for total energy intake, which suggests the intake of snack foods that are rich in fiber [22].

Snack foods that were the top five energy contributors in the present study included both foods that are regarded as health-promoting (e.g., fruits) and foods with a less healthy profile (e.g., sugar/sweets and cakes). Data from NHANES from 2003-2006 showed that the top five sources of snack-derived energy in the US were desserts (including cakes), salty snacks, other snacks, sweetened beverages and fruits/juices [1]. A nationwide dietary survey in Brazil [5] showed that the food group that consisted of sweets and desserts was the largest contributor to energy intake from snacks. Additionally, in Finland, the sweet bakery goods group was one of the top energy-contributing food groups from snacks [2]. These lists indicate both similarities and differences in the top five sources of snack-derived energy compared with the Norkost 3 study. However, a common characteristic of each of the lists is that cakes or similar products were the number one energy-contributing snack food, which suggests that different populations share some of the same challenges with respect to snack composition.

Our results demonstrated that participants with a university/college education were more likely to have consumed fruits as part of a snack than participants without such education. This result is in accordance with findings from other countries of higher fruit intakes in groups with a higher socioeconomic status [23,24].

### Snack eating location

International studies have found that food eaten outside of the home tends to have a less healthy profile than food consumed at home [25]. Kwon et al. [26] studied main meals and snacks eaten at home, out-of-home or in institutions by Korean adults (data from 2007-09) and found that snacks eaten at home seemed to contain less energy than snacks eaten out or in institutions, but this was not tested statistically. In a study of 226 US adults, Liu et al. [27] found that eating at work was associated with greater odds of consuming both healthy and unhealthy snacks. We previously studied differences in the composition of dinner meals eaten in different locations [14] and found that at-home dinners were generally healthier than dinners eaten in other eating locations, with the exception of dinners eaten at work. No differences were observed between dinners eaten at work and dinners eaten at home, but the data were limited, as very few dinners had been consumed at work. These findings are similar to the findings reported herein, as snacks eaten at work actually seemed to have a more favorable composition than snacks eaten at home. One explanation may be that snacks consumed at work are planned to a larger extent than snacks consumed in other locations. In addition, many workplaces in Norway provide fruits free of charge. We may also hypothesize that there is less focus on food indulgence at work compared with at home or in other locations. Our results showed that the majority of snacks were consumed at home or at work. Naturally, the impact the snacks eaten in other locations than these have on the overall diet will depend on how frequently snacks are consumed in these other locations. However, the observation that 19% of all snacks were consumed outside home or work implies that this may be important to total dietary intake. The observed differences among snacks consumed in various locations highlight the importance of distinguishing between different out-of-home eating locations when the associations between out-of-home eating and the composition of the diet are studied.

### Strengths and limitations

The major strengths of the Norkost 3 study are the detailed information about foods, portion sizes, meal types and eating location in addition to the relatively large sample size. However, the low participation rate of 37% limits the generalizability of the results. The proportion of participants with a college/university education was higher than in the general population [14]. Because more highly educated individuals are more likely to consume healthier diets than those with less education [28,29], we may assume that more unhealthy snacks might have been reported if the participation rate had been higher. This is also supported by our previous findings that consumption of fruits as snacks was higher in those with a university/college education compared to those without such education [8]. Hence, the role of fruits as snacks may have been overestimated in the present study due to the high percentage of participants with a college/university education. Nevertheless, fruit was also one of the top five energy-contributing snack foods in participants without a university/college education, which indicates that fruits are important constituents of snacks in populations with a lower education level (data not shown). It is generally recognized that dietary assessment methods that involve self-reports of food intake tend to underestimate food and nutrient intake [30]. The 24 h recall method relies on the ability and willingness of the participants to correctly inform the interviewer of all eating and drinking events that occurred on the preceding day. There is some evidence that snacks are more likely to be underreported than main meals [31]. If this was the case in our study, the impact of snacks on total dietary intake would be larger than what we observed. However, the probing questions that were asked after each of the 24 h recalls were meant to remind the participant of easily forgotten food items, which may have reduced the underreporting of snack events. In the Norkost 3 study, BMI was calculated based on self-reported weight and height. It has been shown that self-reported weight is often underreported [32,33]; thus, some of the overweight participants might have been grouped in the normal weight category. This may have contributed to a reduction in the differences between the two groups.

### Practical implications

Snacking is an important part of dietary intake. Hence, the composition of the snacks consumed is important for the total dietary intake, and subsequently for the health and wellbeing of the population. It is therefore essential to promote healthy snack options. Both healthful and less healthful foods were common snack constituents both in the present study and in studies in other populations. Lloyd-Williams et al. [34] studied the potential benefits on cardiovascular deaths of replacing one unhealthy snack per day with a healthy one in the UK population. It was estimated that this replacement would reduce the number of deaths from cardiovascular disease by 6000 per year. This is a quite substantial benefit of a relatively small dietary modification underlining that snack food choices are important to public health. Our results regarding eating location and snack composition may suggest that snacks consumed in other out-of-home eating locations than work may be the snacks most in need of improvement. More information about what influences our choice of snack foods is needed.

## Conclusions

Snacks were consumed by the vast majority of the participants and contributed to approximately one-fifth of their daily energy intake. The primary constituents of snacks were both favorable (e.g., fruits) and less favorable foods (e.g. cakes, sugar/sweets). Snacks eaten at home or at work were generally healthier than snacks consumed during visits to other private households and snacks consumed at restaurants. Nutritional educators should recommend healthy snack options and raise awareness of the association between the place of consumption and the composition of snacks.

## Abbreviations

MJ: Megajoule
kJ: Kilojoule
24 h recall: 24 hour recall
BMI: Body mass index
EI: Energy intake
BMR: Basal metabolic rate
NHANES: National Health and Nutrition Examination Survey
US: United States
UK: United Kingdom
